# Dynamic [18F]FET-PET/MRI using standard MRI-based attenuation correction methods

**DOI:** 10.1007/s00330-018-5942-9

**Published:** 2019-01-11

**Authors:** Ivo Rausch, Andreas Zitterl, Neydher Berroterán-Infante, Lucas Rischka, Daniela Prayer, Matthias Fenchel, Reza A. Sareshgi, Alexander R. Haug, Marcus Hacker, Thomas Beyer, Tatjana Traub-Weidinger

**Affiliations:** 1 QIMP Team, Center for Medical Physics and Biomedical Engineering, Medical University of Vienna, Vienna, Austria; 2 Division of Nuclear Medicine, Department of Biomedical Imaging and Image-guided Therapy, Medical University of Vienna, Waehringer Guertel 18-20, 1090 Vienna, Austria; 3 Department of Psychiatry and Psychotherapy, Medical University of Vienna, Vienna, Austria; 4 Division of Neuroradiology, Department of Biomedical Imaging and Image-guided Therapy, Medical University of Vienna, Vienna, Austria; 5 Magnetic Resonance, Siemens Healthcare GmbH, Erlangen, Germany; 6 Division of Radiology-Technique, University of Applied Science, Vienna, Austria

**Keywords:** Brain neoplasms, Positron emission tomography, Magnet resonance imaging, Radionuclide imaging

## Abstract

**Aim:**

To assess if tumour grading based on dynamic [18F]FET positron emission tomography/magnetic resonance imaging (PET/MRI) studies is affected by different MRI-based attenuation correction (AC) methods.

**Methods:**

Twenty-four patients with suspected brain tumours underwent dynamic [18F]FET-PET/MRI examinations and subsequent low-dose computed tomography (CT) scans of the head. The dynamic PET data was reconstructed using the following AC methods: standard Dixon-based AC and ultra-short echo time MRI-based AC (MR-AC) and a model-based AC approach. All data were reconstructed also using CT-based AC (reference). For all lesions and reconstructions, time-activity curves (TACs) and time to peak (TTP) were extracted using different region-of-interest (ROI) and volume-of-interest (VOI) definitions. According to the most common evaluation approaches, TACs were categorised into two or three distinct curve patterns. Changes in TTP and TAC patterns compared to PET using CT-based AC were reported.

**Results:**

In the majority of cases, TAC patterns did not change. However, TAC pattern changes as well as changes in TTP were observed in up to 8% and 17% of the cases when using different MR-AC methods and ROI/VOI definitions, respectively. However, these changes in TTP and TAC pattern were attributed to different delineations of the ROIs/VOIs in PET corrected with different AC methods.

**Conclusion:**

PET/MRI using different MR-AC methods can be used for the assessment of TAC patterns in dynamic [18F]FET studies, as long as a meaningful delineation of the area of interest within the tumour is ensured.

**Key Points:**

*• PET/MRI using different MR-AC methods can be used for dynamic [18F]FET studies.*

*• A meaningful segmentation of the area of interest needs to be ensured, mandating a visual validation of the delineation by an experienced reader.*

## Introduction

Positron emission tomography (PET) is a well-established tool for tumour imaging in neuro-oncology. PET imaging using amino acid tracers has been demonstrated to be of particular value for the diagnosis, prognosis and therapy response assessment of glioma patients [1–3]. Moreover, amino acid PET is used for target definition in radiation therapy and for guided surgical biopsy [4, 5]. Among the available amino acid tracers, [18F]2-fluoroethyl-l-tyrosine ([18F]FET) is one of the most frequently used. Several studies have shown the value of [18F]FET for the assessment of gliomas [1, 6–9]. Moreover, its clinical adoption has been recently recommended by the Response Assessment in Neuro-Oncology Working Group together with the European Association for Neuro-Oncology [2]. Particularly, the observed [18F]FET uptake behaviour within a brain tumour lesion over the time evaluated by different PET techniques enables a better understanding for brain tumour grading with the possibility to detect anaplastic foci and treatment response [10–13].

These evaluation techniques are based on a classification of the tracer uptake over time into two [14] or three [15] curve patterns and have shown to increase the diagnostic accuracy in relatively large patient cohorts in stand-alone PET and PET/CT examinations.

With the introduction of fully integrated PET and magnetic resonance imaging (PET/MRI), a new modality has become available, which has the potential to improve the diagnostic accuracy compared to PET or PET/CT imaging. The MRI component not only provides a high-resolution and high-contrast anatomical reference for the tracer distribution but also helps probe cellular and molecular pathways through advanced MRI techniques, such as diffusion-weighted imaging, perfusion imaging and spectroscopy [16, 17]. These advanced MRI techniques enable a more detailed soft tissue analysis and showed promising results for an improvement in discriminating malignant from benign lesions and glioma grading [18, 19]. However, only few studies have been published regarding the impact of combined PET/MRI in the clinical management of glioma patients [19, 20] and PET/MRI has primarily been suggested to be beneficial especially in paediatric patients [21]. In this patient group, the reduced radiation burden arising from the CT component in PET/CT examinations is particularly desirable. Further, general anaesthesia is often required for paediatric patients for MRI as well as PET. Performing both examinations simultaneously in one PET/MRI session has the advantage of reduced risks related to anaesthesia in this group of patients.

Nevertheless, clinical PET/MRI is still challenged by technical issues related to attenuation correction (AC) [22]. Reasons are that MRI information is not related to the attenuation properties of the investigated tissue [23]. Further, the visualisation of bone is challenging in MRI, and thus, standard MRI-based AC (MR-AC) methods do not account for bone, which has been shown to yield a regionally variable bias in the reconstructed tracer distribution [24]. Several MR-AC methods have been developed to overcome this issue [25]. However, for clinical routine, just three methods are available in current PET/MRI systems. These include the standard Dixon-based MR-AC method, an ultra-short echo time (UTE)-based method and a model-based approach [26–29]. The quantitative accuracy of these MR-AC methods has been assessed for static PET examinations and standardised uptake values (SUVs) [22, 24, 25, 28, 29]. However, none of these studies assessed the influence of the available MR-AC methods on the dynamic evaluation of [18F]FET examinations by means of a categorisation of the uptake pattern into different pattern classes. On first glance, one could assume that a bias in quantification only results in a scaling of the tracer uptake patterns but does not change the shape, and therefore, the categorisation of the patterns itself. However, as the above-mentioned studies revealed spatially variant biases in quantitative readings and the uptake patterns are extracted from regions selected based on the tracer distribution, a straightforward translation of the established techniques to PET/MRI is probably not possible.

Therefore, the aim of our study was to evaluate if the established classification of uptake curves of dynamic [18F]FET PET imaging of glioma patients is applicable to PET/MRI data using different MR-based attenuation correction techniques.

## Materials and methods

### Subjects and study protocol

This study included 24 patients (20 female, mean age ± SD = 49 ± 14 years) referred for a [18F]FET-PET examination due to findings in earlier performed MRI examinations. These findings included suspected primary (*n* = 5) and recurrent (*n* = 11) gliomas as well as suspected metastasis (*n* = 8). For further details, see Table 1. All patients underwent a PET/MRI examination of the brain (Biograph mMR, Siemens Healthineers) starting with a bolus injection of about 200 MBq [18F]FET. PET emission data were acquired in listmode for 40 min. The protocol included the acquisition of the following MR sequences: standard Dixon, standard UTE and an anatomical T1-weighted MRI of the brain (MP-RAGE, TE = 4.2 ms, TR = 2000 ms, voxel size = 1 mm × 1 mm × 1 mm). Following the PET/MR examination, an additional low-dose CT of the head was acquired on a whole-body PET/CT system (Biograph TPTV, Siemens Healthcare GmbH) for the purpose of CT-based attenuation correction (CT-AC). This study was approved by the Ethics Committee of the Medical University of Vienna (EK-No. 1828/2016). All patients did give their written informed consent before the examination.

**Table 1:** Overview of patients included in this study. The given diagnosis was based on the latest available histological findings except for two patients with suspected low grad glioma

Patient	Age	Sex	Indication for 18F[FDG] PET/MR	Therapy before PET/MR	Diagnosis based on histology	Localisation
1	22	F	Primary	None	Anaplastic astrocytoma II–III*	Frontotemporal left
2	43	M	Recurrent	OP, CH, RT, GKN	Anaplastic astrocytoma III	Temporal left
3	37	F	Primary	None	Not established (suspected low grad glioma)	Operculum right
4	52	F	Recurrent	RT	Diffuse astrocytoma II	Pons
5	51	F	Recurrent	CH, RT	Oligodendroglioma II	Temporal left
6	27	F	Primary	None	Not established (suspected low grad glioma)	Thalamus left
7	53	F	Metastasis	CH, RT,GKN	Breast carcinoma*	Frontal right
8	38	M	Primary	None	Diffuse astrocytoma II*	Frontal left
9	44	F	Recurrent	OP	Oligodendroglioma II	Frontal left
10	69	F	Metastasis	CH, RT, GKN	Breast carcinoma	Cerebellar right
11	34	F	Recurrent	CH, RT	Diffuse fibrillar astrocytoma II–III	Basal ganglia right
12	55	F	Metastasis	OP, RT, CH, GKN	Cervix carcinoma*	Frontal left
13	37	F	Recurrent	OP, RT, CH	Glioblastoma	Frontal left
14	26	F	Recurrent	OP	Diffuse astrocytoma II	Parietal right
15	63	F	Primary	None	Oligodendroglioma II*	Temporal left
16	45	F	Recurrent	OP	Diffuse astrocytoma II–III	Parietal right
17	59	F	Recurrent	OP, RT, CH	Anaplastic oligodendroglioma III	Frontal left
18	75	M	Metastasis	CH, ST, GKN	Lung carcinoma	Frontal left
19	51	F	Metastasis	OP, CH, GKN	Lung carcinoma	Temporal, occipital right
20	53	F	Recurrent	OP, RT	Anaplastic oligoastrocytoma III	Frontal, temporal right
21	46	F	Recurrent	OP, CH	Anaplastic oligoastrocytoma II–III	Frontal right
22	67	F	Metastasis	CH, GKN	Breast carcinoma	Parietal right
23	66	F	Metastasis	CH, GKN	Breast carcinoma	Frontal right
24	64	F	Metastasis	CH, GKN	Melanoma	Cerebellar right

*F* female, *M* male, *OP* surgery, *RT* radiation therapy, *CH* chemotherapy, *GKN* gamma-knife therapy

*Histology was established after PET/MR examination

### Data processing

**Fig. 1:**
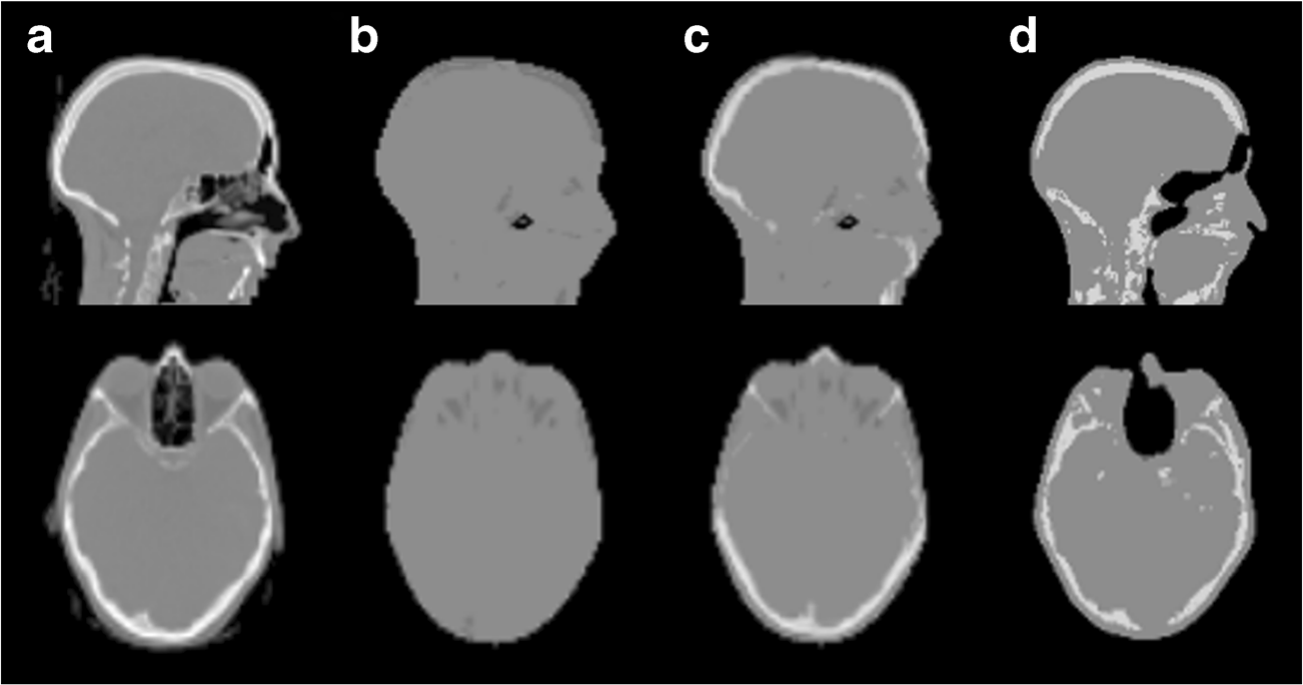
Example of the used MR-AC methods compared to CT-AC. **a** CT-AC. **b** Dixon-AC. **c** Model-based AC. **d** UTE AC. Image shows the same sagittal and axial slices of one patient

For each acquisition, the 40-min dynamic PET data were reconstructed into eight frames (2 × 2 min, 4 × 4 min, 2 × 10 min) using four different attenuation corrections: standard Dixon-based MR-AC (Dixon-AC) [26], standard UTE-based MR-AC (UTE-AC) [28], model-based MR-AC and CT-AC [29, 30] (Fig. 1). In short, the Dixon-AC is based on the Dixon technique [31] and results in a four-class AC map with uniform linear attenuation coefficients for soft tissue, fat, air and, if applicable, lung [27]. The UTE-AC is based on an ultra-short echo time sequence enabling the generation of an AC map categorising the head into three tissue classes (soft tissue, bone and air) [28]. Finally, the model-based AC incorporates spatially variant attenuation coefficients of the major bone structures into the Dixon-AC [29, 30]. It employs a prototype version of the five compartment AC method available for the mMR PET/MRI system in software version E11.

We use the images following CT-AC as a reference standard. For the CT-AC, the low-dose CT acquired with the PET/CT was co-registered rigidly to the anatomical T1-weighted MRI images after removal of the patient bed from the PET/CT. Then, the CT image volume was resampled to the spatial resolution of the Dixon-AC map and the Hounsfield units were converted into linear attenuation values for 511 keV photons using the same segmentation/bi-linear scaling approach as used in the PET/CT system [32].

All image reconstructions were performed on an external workstation (e7tools, Siemens Healthcare GmbH) using an ordinary Poisson ordered-subset expectation maximisation (OP-OSEM) algorithm with the following image reconstruction settings: 3 iterations, 21 subsets, matrix size = 172 × 172, zoom factor = 2 and post-reconstruction filter = 3 mm FWHM Gaussian kernel.

### Data evaluation

The evaluation of the dynamic [18F]FET studies was based on the most common evaluation approaches as described by Jansen et al [10, 11] and Pöpperl et al [14, 33] and Galldiks et al [15, 34]. In general, all methods followed the same concept. First, a summed PET image is generated from the last 20 min of the acquisition (21–40 min post tracer injection). Then, tumours are delineated using region of interest (ROI) or volume of interest (VOI). In this work, ROI refers to a connected area in one single image slice. VOI refers to a connected volume, which can include connected voxels from multiple slices. ROIs and VOIs are copied to all time frames of the dynamic PET data, and time-activity curves (TACs) are extracted from this data. TACs are composed of the average SUV of all voxels in a ROI or VOI within a reconstructed frame and the time elapsed from the start of the acquisition to the end of the respective frame. These TACs are then categorised by their shapes into different TAC patterns, which are linked to differences in expected overall survival time or represent different tumour grades [14, 15]. The differences between the methods used by different groups are found in the ROI or VOI definition and in the pattern categories.

In this study, we replicated the most common evaluation techniques published by Jansen et al [10, 11] and Pöpperl et al [14] as well as Galldiks et al [15, 34]. In accordance with their published methodology, the following ROI and VOI definitions and pattern categories combinations were used:

- ROI_90_: ROI in the axial slice with the highest uptake in the tumour generated by a threshold-based region growing including all (surrounding and connected) voxels whose SUVs were equal or greater than 90% of the of the maximum SUV within the tumour
- VOI_90_: Similar to ROI_90_, but in three dimensions

Similar to Jansen et al [10] and Pöpperl et al [14], TACs extracted using ROI_90_ and VOI_90_ were categorised into the following [18F]FET uptake patterns:

(I) Increasing, with [18F]FET uptake constantly increasing, and
(II) Decreasing, with TAC reaching a maximum within 20 min, followed by a decrease

- ROI_TBR_: ROI in the axial slice with the highest uptake in the tumour generated by a threshold-based region growing including all (surrounding and connected) voxels with SUV equal to or higher than 1.6 times the background tracer concentration. The background uptake is determined as the average tracer concentration in a spherical, 2-cm-diameter VOI placed contralateral on an unaffected brain region (enclosing grey and white matter)
- VOI_TBR_: Similar to ROI_TBR_, but in three dimensions
- VOI_Fix_: Spherical VOI of ~ 2 mL volume (1.56 cm diameter) centred around the maximum tracer uptake in the tumour [15]

Similar to Galldiks et al [15, 34], TACs extracted using ROI_TBR_, VOI_TBR_ and VOI_Fix_ were categorised into the following [18F]FET uptake patterns:

(I) Increasing, with [18F]FET uptake constantly increasing,
(II) Plateau, with TAC reaching a maximum between 20 and 40 min followed by a plateau or slight descent, and
(III) Decreasing, with TAC showing an early peak within 20 min followed by a continuous descent

Lesion definition and TAC extraction were performed using the Hermes Hybrid Viewer software (version 2.6, Hermes Medical). TACs extracted from threshold-based VOIs (VOI_90_, VOI_TBR_), but with the threshold being too low for a meaningful segmentation (i.e. segmentation included large fractions of the neck and the viscerocranium), were excluded from further analysis. For all ROI and VOI definitions, changes in the corresponding TAC categorisation were reported as a function of the MR-AC method.

In addition to the assessment of changes in the TAC categorisation, changes in time to peak (TTP), which is the elapsed time between tracer injection and maximum tracer concentration in the target region within the 40 min post injection, were evaluated for all ROI and VOI definitions and MR-AC methods. Furthermore, differences in the tissue-to-background ratio (TBR) in the last 10-min frame (31–40 min post tracer injection) and threshold-defined volumes in PET images reconstructed with the different MR-AC methods were reported.

To evaluate the sole contribution of the MR-AC on the tracer uptake pattern, TACs were additionally extracted from VOIs placed at exactly the same location for all MR-AC methods. Therefore, an experienced nuclear medicine physician (> 10 years of experience in neuro-oncological imaging) placed spherical VOIs of 1 cm diameter within the 18F-FET avid lesion on the CT-AC reconstructions, thereby avoiding the inclusion of obviously non-malignant structures (e.g. major blood vessels). These VOIs were then copied to the MR-AC PET images, and TACs were extracted. These TACs were categorised according to all above-mentioned methods, and MR-AC-dependent changes in TAC categorisation and TTP were assessed.

## Results

TACs were extracted from ROI_90_, ROI_TBR_ and VOI_Shpere_ for all 24 patients. Using VOI_90_ and VOI_TBR_, TACs were extracted for 23 and 17 patients, respectively. For the remaining patients, VOI_90_ and VOI_TBR_ segmentations included large parts of the head in at least one of the reconstructions and, therefore, were excluded.

**Fig. 2:**
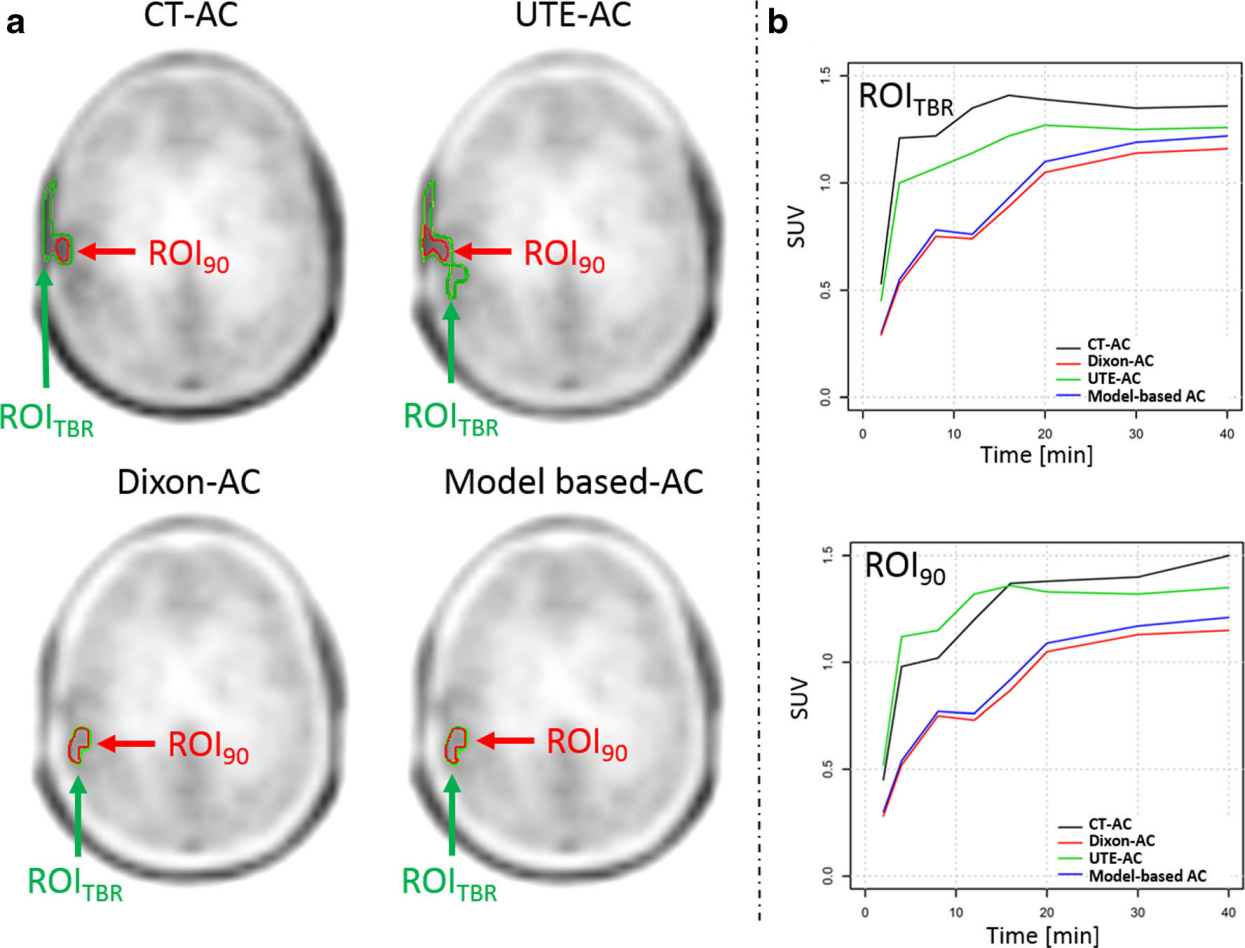
**a** ROI_90_ and ROI_TBR_ delineated on the summed (20–40 min) PET images following three MR-AC and the CT-AC methods. In the CT-AC and UTE-AC PET, the ROI_TBR_ included physiological [18F]FET uptake in the scalp. Further, in the UTE-AC PET, the ROI_TBR_ segmentation resulted in an extended ROI when compared to ROI_TBR_ in the Dixon-AC and model-based AC PET. **b** TAC extracted from ROI_90_ and ROI_TBR_ in PET images corrected using the different AC methods

**Fig. 3:**
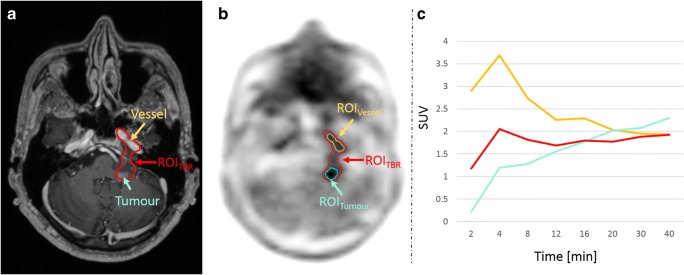
**a** T1-weighted MRI with a contrast-enhancing lesion (cyan arrow) and the arteria carotis interna (orange arrow). **b** ROI_TBR_ delineation of a tumour in the PET image after AC using the model-based approach. In this case, the ROI_TBR_ additionally includes parts of the arteria carotis interna. **c** TACs extracted from ROI_TBR_ (red) and from two manually drawn ROIs in the tumour (ROI_Tumour_, cyan) and the arteria carotis interna (ROI_Vessel_, orange). The TAC extracted from ROI_TBR_ is a mixture of the TAC extracted from ROI_Tumour_ and ROI_Vessel_

No change in TAC pattern was observed for TACs extracted from VOI_90_ and VOI_Fix_. For ROI_90_, ROI_TBR_ and VOI_TBR_ changes in TAC pattern categorisations were observed in up to 4%, 8% and 6% of the examinations when using different MR-AC methods, respectively. Table 2 summarises the percentage of changes in TAC categories depending on the choice of the MR-AC method in relation to the reference standard (CT-AC). In cases where TAC categorisation did change, differences in the delineated ROI/VOI by means of their volume and location could be observed (Figs. 2 and 3). TTP changes were found for all ROI/VOI definitions in a varying percentage of cases depending on the used MR-AC method (Table 3).

**Table 2:** Changes (% total) of TAC categories derived from ROI and VOI analysis of PET data following MR-AC as compared to CT-AC

AC method	2 TAC pattern categories		3 TAC pattern categories		
	ROI_90_ (%)	VOI_90_ (%)	ROI_TBR_ (%)	VOI_TBR_ (%)	VOI_Fix_ (%)
Dixon	0	0	8	6	0
UTE	4	0	4	0	0
Model-based	0	0	8	6	0

**Table 3:** Number of patients in relation to the total number of evaluated patients where TTP derived from ROI and VOI analysis of PET data changed following MR-AC as compared to CT-AC

AC method	ROI_90_	VOI_90_	ROI_TBR_	VOI_TBR_	VOI_Fix_
Dixon	2/24 (1, 2)	0/23	3/24 (1, 1, 3)	1/17 (1)	1/24 (1)
UTE	2/24 (1, 3)	4/23 (1, 1, 1, 1)	3/24 (1, 1, 1)	1/17 (2)	2/14 (1, 1)
Model-based	2/24 (1, 1)	1/23 (1)	4/24 (1, 2, 3, 6)	2/17 (2, 6)	2/24 (1, 1)

The numbers in brackets are the ΔTTP in units of time frames for the cases where TTP changed (e.g. (1,3) means TTP shifted one time frame in one case and three time frames in another)

**Fig. 4:**
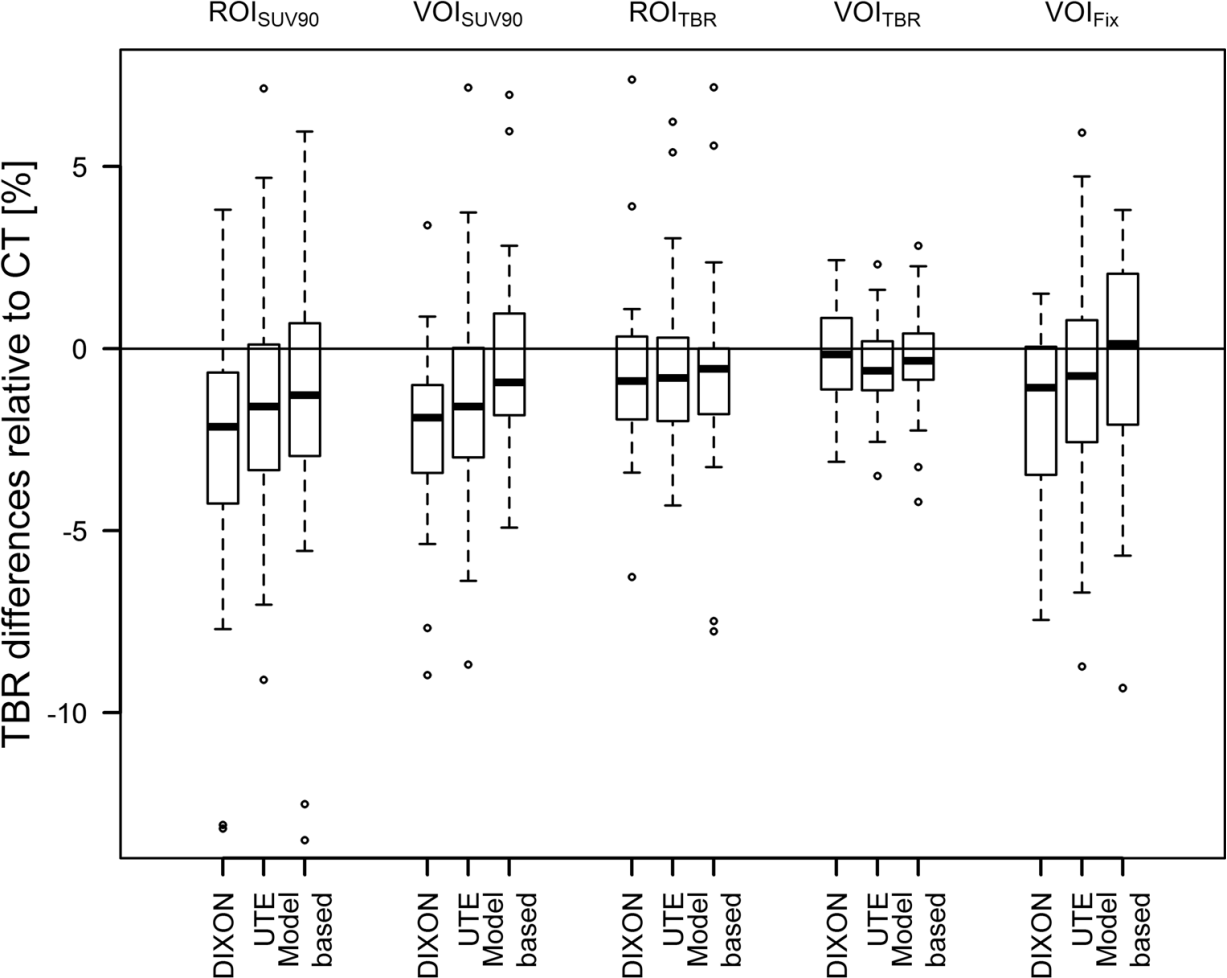
Relative difference of TBRs using the three MR-based AC methods in comparison to the TBRs extracted from PET after CT-AC

**Fig. 5:**
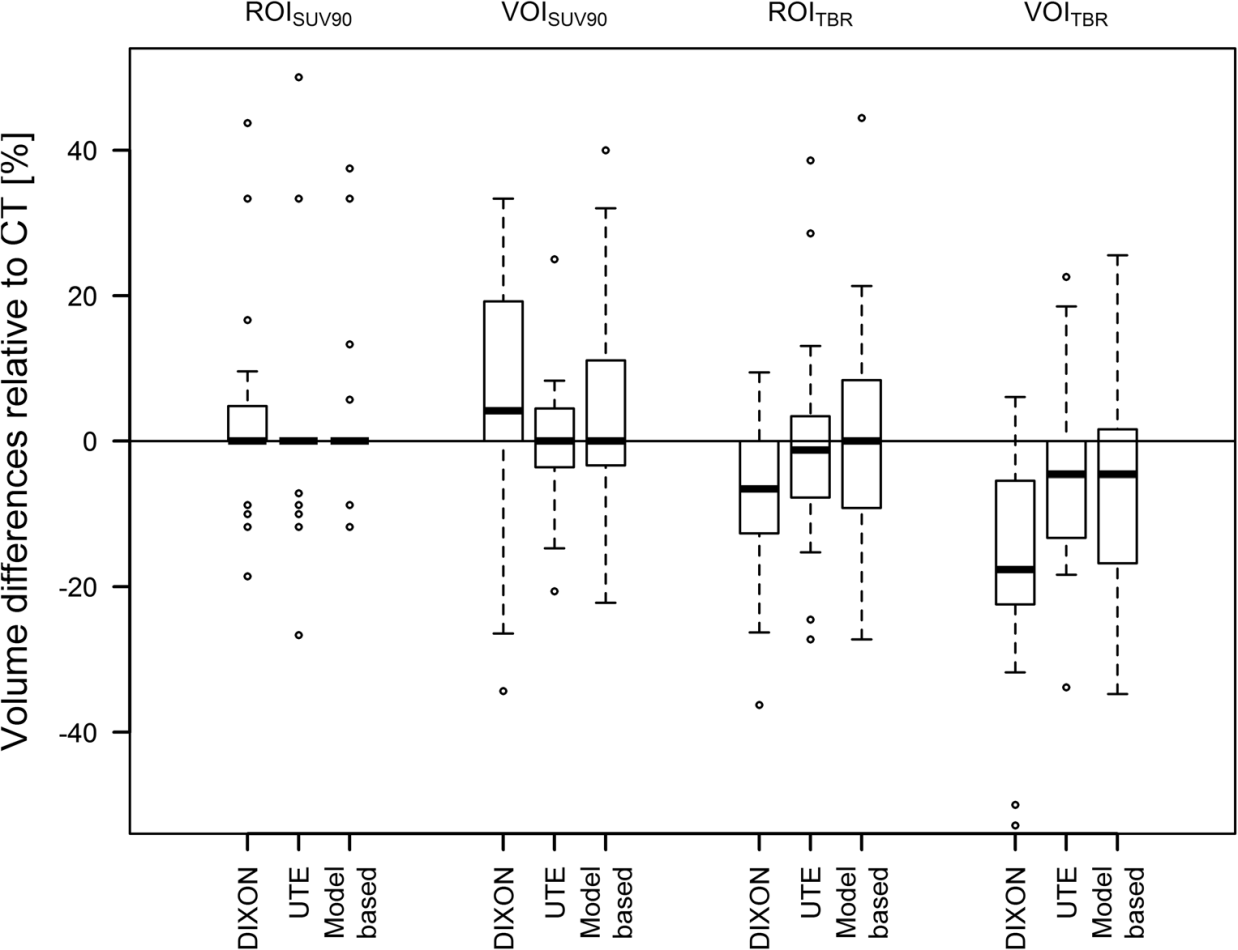
Relative difference of the PET-based ROI and VOI for the three MR-AC methods in comparison to the ROI and VOI following CT-AC

TBRs were similar for all AC methods and ROI/VOI definitions. Average deviations of TBRs compared to TBRs after CT-AC PET were between 0 and − 3%, depending on the ROI/VOI definition and MR-AC method. Figure 4 depicts the changes in TBRs compared to CT-AC PET. The median absolute delineated volumes in the CT-AC PET where 0.1 mL, 0.6 mL, 0.3 mL and 4 mL for ROI_90_, ROI_TBR_, VOI_90_ and VOI_TBR_, respectively. Average changes of volumes of the ROIs/VOIs in relation to CT-AC PET were between + 8% and − 17% when using different MR-AC methods and ROI/VOI definitions with maximum relative changes of ROI/VOI volumes of up to − 63% (ROI_TBR_, model-based AC) and + 138% (ROI_90_, model-based AC) (Fig. 5). However, changes in absolute delineated volumes > 1 mL only occurred for VOI_TBR_-based delineations in five cases.

The evaluation of the sole MR-AC-based contribution on the uptake pattern categorisation revealed that for the exactly similar located VOI, no changes in pattern categorisation and TTP were present.

## Discussion

This study evaluated if TAC pattern categorisations of dynamic [18F]FET PET studies are influenced by the use of MR-AC in PET/MRI imaging. It was shown that in the majority of cases, no change in TAC pattern categorisation occurred when using different MR-AC methods and that tumour grading in [18F]FET PET seems to be feasible, regardless of the choice of the MR-AC method.

In general, local biased quantitative readings (e.g. SUVs) are known to occur in PET after MR-AC when compared to reference CT-AC [22, 24, 25]. Such bias results from known limitations of MR-AC, such as incorrect assumptions of local tissue attenuation [25], and thus, to locally biased readings of the attenuation-corrected emission data. However, in dynamic emission studies, all time frames are corrected using the same AC map. Thus, a relative quantitative bias in a certain location should be stable for all reconstructed time frames as long as movement artefacts can be neglected. Such a systematic error will lead to a scaling of the quantitative readings but should not influence the shape of a TAC for a given ROI or VOI. Furthermore, any quantitative bias in brain PET/MRI is approximately symmetrical across both brain hemispheres [22, 29]. Therefore, TBR should be similar between different AC methods when sampling the background tracer uptake contralateral to the evaluated ROI or VOI. These assumptions are also confirmed by our results, where similar TBR values are found for all AC methods (Fig. 4) and no changes in categorisation and TTP were present for the TACs extracted from the VOIs placed at exactly the same location for all AC methods.

However, in this study, TAC pattern changes as well as shifts in time of the TTP, when using established methodology with different MR-AC methods, were observed in up to 8% and 17% of the cases, respectively. These changes are attributed to differences in the segmented ROI or VOI. Threshold-based region growing segmentations depend on the set threshold value and on the local tracer distribution. A locally varying bias of PET activity, as observed in PET images after MR-AC [24], may affect the representation of a local tracer distribution and, thus, contributes to differences in the segmented volume (Fig. 2). These volumes also represent diverging investigated areas and, thus, distinct tissues with individual uptake behaviours. Furthermore, another segmented area may also represent a tumour area with an altered uptake pattern, given the possibility of a heterogeneous tumoural lesion.

In addition to altered segmentations within a lesion, obviously wrong segmentations were found in the evaluated datasets. Such errors in segmentations were most prominent when the tumour was located near other anatomical structures with physiological [18F]FET accumulation (e.g. blood vessels or scalp). In such cases, the threshold-based segmentation methods could not always distinguish increased [18F]FET uptake of a tumour from the increased uptake of non-tumoural tissue reliably. Figure 3 shows an example where a blood vessel was included in the VOI_TBR_ following model-based MR-AC. As a result, the TAC was a mixture of the blood TAC (input function) and the TAC of the lesion. In this case, an exclusion of the blood vessel would have resulted in similar TAC patterns for all AC methods. Such issues are an inherent property of semiautomatic thresholding algorithms. Therefore, an incorrect tumour segmentation cannot be attributed to a specific AC method and, thus, can also occur after CT-AC.

In general, segmentations with high thresholds (e.g. a threshold of 90% of the maximum uptake) appear to be more stable by means of changes of the segmented region. However, the differences in bias distribution from different MR-AC methods can lead to a change in the location of the maximum uptake in a lesion. This will lead to a change of the starting point of the region growing and, again, will result in a different delineated area. The differences found for TTP extracted from the VOI_Fix_ are also partly attributed to changes of the maximum pixel location. Moreover, TTP changes could be caused by small adjustments by the experienced reader when manually placing the VOI_Fix_, thus reflecting intra- and inter-reader variabilities. Nevertheless, for the categorisation of TACs, VOI_Fix_ showed the most stable behaviour (Table 2) and the exclusion of obviously non-tumoural tissue could be ensured by the experienced reader.

A change in the appearance of the local tracer distribution and the location of the maximum uptake in a lesion could potentially also influence [18F]FET PET-based targeted biopsy [1, 5, 13] or radiotherapy planning [35]. Especially, in radiation therapy planning, threshold-based delineation approached are often used [36, 37]. Here, similar changes of delineated volumes for different MR-AC concepts as found in this study can be expected. These variabilities of volumes might be of minor impact for high threshold values (e.g. VOI_90_), due to the small absolute volume changes (< 1 mL). However, for low threshold segmentations (e.g. VOI_TBR_), such volume changes might be significant.

In this study, the used methods for tumour segmentations were replications of published methodologies for which the diagnostic value of dynamic [18F]FET PET was proved in large patient cohorts for stand-alone PET examinations [10, 14, 15, 34]. These methods are based on region growing algorithms applied to PET images (except the VOI_Fix_) and do not take into account a potential involvement of structures with physiological uptake. Therefore, the definition of the region of interest should not be purely based on PET images. Especially in the case of PET/MRI, where co-localised anatomical information is available, this information should be additionally utilised for the delineation of the targeted structures (Fig. 3).

However, even with purely PET-based delineation methods, only a small proportion of the evaluated TAC patterns were categorised differently when using different MR-AC methods. TACs extracted from fixed VOIs and threshold-based segmentations with conservatively high thresholds only defining the most metabolic active areas showed the most stable behaviour. Segmentations based on relatively low thresholds (e.g. 1.6 times the background) aiming on a segmentation of the total tumour volume were more likely to result in different TAC patterns. However, none of the investigated delineation methods has shown a 100% reproducible segmentation of the investigated area in all cases.

### Limitations

The study protocol did not include motion correction of the dynamic PET data. Solely foam wedges were used to limit subject motion. Therefore, motion artefacts cannot be ruled out. However, no influence of motion artefacts on the results of the pattern comparison is expected as the same raw data was used in all reconstructions, and thus, an artefact will influence all reconstructions similarly. Nevertheless, motion compensation is highly recommended for dynamic studies as motion artefacts could influence the pattern categorisation.

In this study, a high prevalence of lesions with relatively low tracer uptake was present. We assume that the rate of failing segmentations using VOI_90_ and VOI_TBR_ is lower in patient cohorts with higher tracer uptake in the lesions.

## Conclusion

Established uptake curve evaluation methods used for glioma characterisation in dynamic [18F]FET PET can be applied to PET/MRI using all investigated MR-AC techniques. However, quantitative readings by means of SUV may be biased depending on the used MR-AC. Moreover, a meaningful segmentation of the area of interest needs to be ensured with all AC methods. This mandates a visual validation of the ROI or VOI by an experienced reader, preferably also using the additional anatomical information as provided by the MRI component of the PET/MRI system.

## Abbreviations

AC: Attenuation correction
CT-AC: CT-based attenuation correction
MR-AC: MRI-based attenuation correction
ROI: Region of interest
SUV: Standardised uptake value
TAC: Time-activity curve
TBR: Tissue-to-background ratio
TTP: Time to peak
UTE: Ultra-short echo time
VOI: Volume of interest
